# Health Tract, No. 153

**Published:** 1864-11

**Authors:** 


HEALTH TRACT, No. 153.

### BILIOUS DIARRHEA

Is always an effort of nature to save herself from impending disease; hence it is a curative process, and should not be interfered with. The passages in dysentery are bloody and painful always; in simple diarrhea they are always thin, almost watery, always large and light colored. In cholera, which is aggravated diarrhea, the passages are infallibly painless; on the other hand, bilious diarrhea is known by the passages being colored either dark, green, or yellow, often with a burning, griping, or other ill feeling before the passages come on. Bilious diarrhea ought never to be checked, except by medical advice, because it is an effort of nature to rid the system of that which would destroy it, if allowed to remain within it. Life has been destroyed thousands of times by failing to distinguish a bilious diarrhea from common diarrhea, simply by not noticing the color of the discharges, and thinking that nothing more is necessary than to "check it;" and that whatever does this the quickest is the best remedy. Opium, and paregoric, and laudanum, and morphine are resorted to with a fatal recklessness; they arrest, but they do not cure; they hide, cover over, but do not eradicate; but that is not the worst of it, they often send the disease to the brain, especially in children, to result in certain death in a short time. In most cases, all that is necessary in bilious diarrhea is to take nothing, keep still, keep warm in bed, and do not eat an atom of any thing, except when really hungry. There is but a step between bilious diarrhea and bilious or cramp colic, these last ending in death often within a few hours. The difference between them is only this—nature forces the bile out of the system in bilious diarrhea; in bilious colic she has not strength to do it, and in this latter case, unless speedily and efficiently aided, death, painful, agonizing and speedy, is the result.

Bilious diarrhea is often preceded by costiveness, and is generally brought on by bad air or by chilling the skin, either by cooling it off too soon after exercise, or by remaining in water or damp garments for a long time; the effect in either case is the same, to wit, to close the pores of the skin and drive the matters back and inward, which would otherwise have escaped beneficially from the body. A sudden burst of passion or other shock or great mental emotion may bring on an attack of bilious diarrhea. Those, therefore, who have observed themselves to be subject to attacks of bilious diarrhea, may easily postpone them indefinitely by arranging to have the bowels act freely once every twenty-four hours, by cultivating an equable frame of mind, and by habitually avoiding every thing which causes a chilly feeling to the skin; for he is not the greatest man who can the most readily cure diseases in others, but he who is most successful in preventing them in his own person.

